# Tissue context as a determinant of trained innate immunity: a conserved molecular toolkit with divergent functional outputs

**DOI:** 10.1093/jleuko/qiag047

**Published:** 2026-04-09

**Authors:** Mary A Oliver, Xenia D Davis, Julia K Bohannon

**Affiliations:** Department of Pathology, Microbiology and Immunology, Vanderbilt University Medical Center, 1211 Medical Center Drive, Nashville, TN 37232, United States; Department of Anesthesiology, Vanderbilt University Medical Center, 1211 Medical Center Drive, Nashville, TN 37232, United States; Department of Pathology, Microbiology and Immunology, Vanderbilt University Medical Center, 1211 Medical Center Drive, Nashville, TN 37232, United States; Department of Anesthesiology, Vanderbilt University Medical Center, 1211 Medical Center Drive, Nashville, TN 37232, United States

**Keywords:** epigenetics, immunometabolism, tissue-resident macrophage, trained innate immunity

## Abstract

Trained innate immunity (TI) challenges the traditional view that adaptive immune cells are solely responsible for establishing immune memory. Instead, innate immune cells can develop a form of memory through persistent epigenetic, metabolic, and antimicrobial modifications, enabling them to respond to secondary challenges in a nonspecific manner. While the molecular mechanisms underlying this trained response have been extensively characterized and are well understood, the intrinsic cellular programs driving trained immunity have not been clearly delineated. Further, the influence of tissue-specific microenvironments remains underexplored. Evidence indicates that the heterogeneity observed in trained immune responses is partly attributable to the functional outcomes shaped by trained immunity within diverse tissue microenvironments, underscoring the complexity and context-dependent nature of this adaptive process.

In this review, we explore that TI uses a conserved molecular toolkit whose functional output is dictated by tissue microenvironment. Signals such as oxygen tension, microbiota, local metabolites, cytokine release, and damage-associated molecular patterns can also shape trained innate immunity. The resulting outcomes range from increased antimicrobial defense to maladaptive responses that lead to chronic inflammation and tissue damage. Together, we synthesize findings from hematopoiesis and tissue-resident macrophage biology, emphasizing how immunometabolism and epigenetic mechanisms underpin tissue-specific models of TI. This comprehensive framework resolves contradictions observed across different organs and disease states, positioning tissue instruction as a pivotal determinant of innate immune memory. It demonstrates that trained immunity programs are intricately adapted to tissue niches, with profound implications for infection control, inflammatory diseases, tissue regeneration, and the precise therapeutic targeting of innate immune cells.

Key Concepts

- Conserved Molecular Basis of TI
- Tissue microenvironment is a key determinant of TI outcomes
- Integration of progenitor-level and tissue-resident training
- Implications for disease and therapeutic targeting

Open Questions

- How conserved is TI across tissue microenvironments?
- What are the relative contributions of progenitor versus tissue-resident training?
- Are TI states stable or reversible within tissues?

## 1. Introduction: trained innate immunity—from classical to tissue-specific modulation

**Figure 1:**
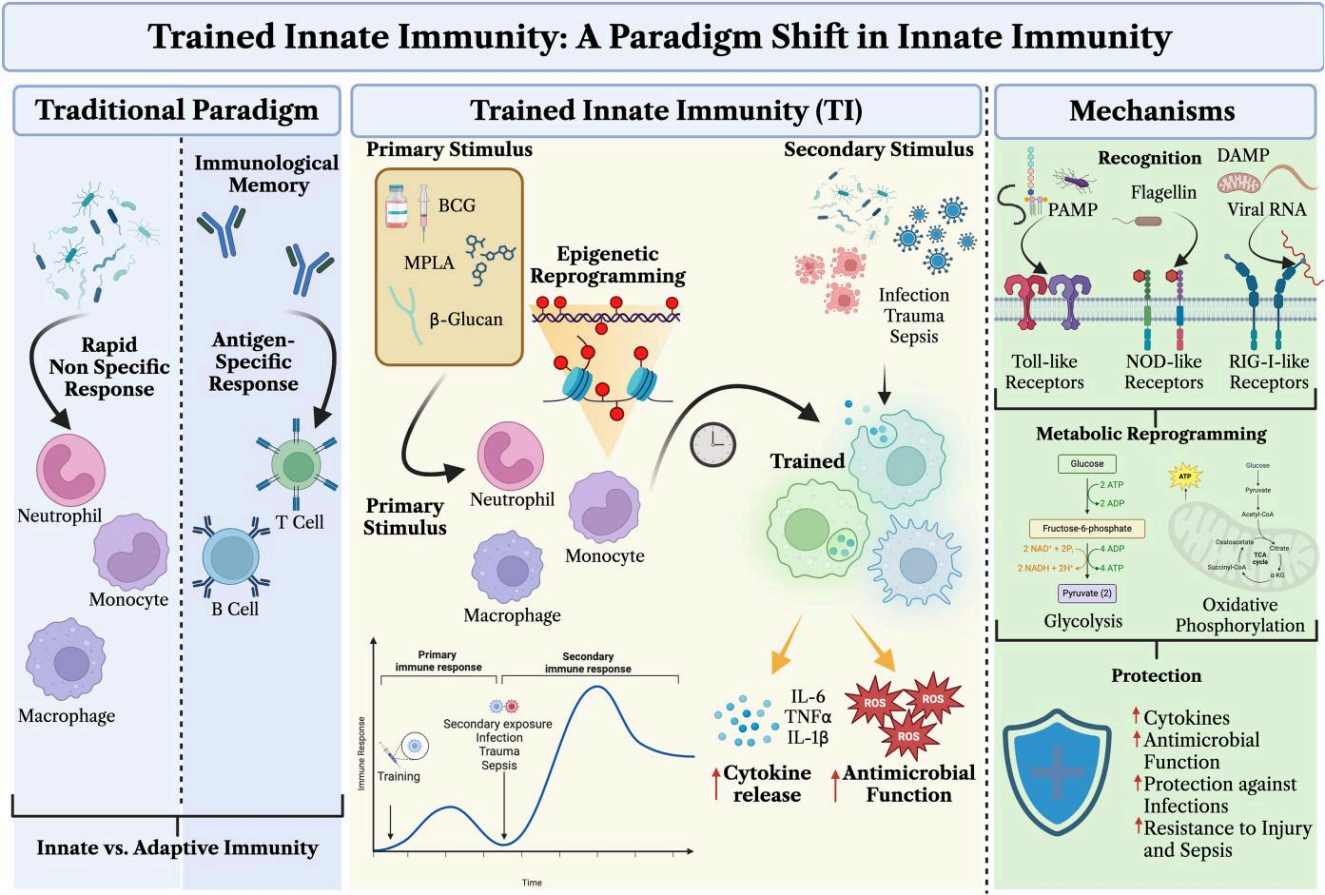
Classical trained innate immunity (TI). Traditionally, innate immune responses have been considered rapid, nonspecific, and lacking memory, as innate cells are short-lived, with lifespans ranging from a few hours to days. Conversely, adaptive immunity is well-established as the memory component of the immune system, exhibiting antigen-specific memory responses mediated by T cells and B cells against foreign and pathogenic agents. However, TI challenges this traditional view by demonstrating that innate immune cells can develop nonspecific memory-like responses following primary stimulation with agents such as BCG, MPLA, or β-glucan. Epigenetic reprogramming and metabolic rewiring—characterized by enhanced glycolysis or oxidative phosphorylation—lead to a sustained heightened immune response in innate cells. Upon secondary exposure, these cells have shown protective effects against infection, trauma, and sepsis. This form of training is mediated through various pattern recognition receptors, including TLRs, NOD-like receptors, and RIG-I-like receptors. Collectively, these mechanisms redefine innate immunity, illustrating that the innate immune system is capable of functional enhancement that results in long-lasting immunological memory. Created with Biorender.com.

Traditionally, innate immunity has been characterized by rapid, nonspecific responses to injury or infection, with the generation of antigen-specific responses and immunological memory regarded as hallmarks of adaptive immunity. Recent groundbreaking discoveries, however, have fundamentally challenged this dichotomy by revealing that innate immune cells can exhibit a form of memory, termed trained innate immunity (TI). TI involves a primary stimulus that epigenetically reprograms innate immune cells, thereby enhancing their responsiveness to subsequent challenges in a robust and long-lasting manner (Fig. 1). Initial investigations by Netea et al.^1^ introduced the concept of trained immunity, demonstrating that innate immune cells exhibit heightened, nonspecific responses upon secondary stimulation. Early studies corroborating these findings showed that Bacillus Calmette-Guérin (BCG) vaccination and beta-glucan (β-glucan) exposure induce elevated cytokine production, modulate myelopoiesis, and activate antimicrobial mechanisms not only in mature immune cells but also in hematopoietic stem and progenitor cells (HSPCs).^2–7^ Moreover, such reprogramming confers protective effects against lethal infections, as evidenced by BCG's capacity to safeguard severely immunodeficient SCID mice from *Candida albicans* through NOD2-mediated pathways.^8^ Many pattern recognition receptors (PRRs) can induce trained immunity, including toll-like receptors (TLRs), nucleotide-binding oligomerization domain-like receptors (NLRs), and RIG-I-like receptors (RLRs), among others. These PRRs detect microbial components known as pathogen-associated molecular patterns (PAMPs) and DAMPs, triggering antimicrobial responses and metabolic reprogramming that confer protective effects upon subsequent stimuli (Fig. 1). Notably, key PAMPs such as lipopolysaccharide (LPS) derivative and TLR4 agonist monophosphoryl lipid A (MPLA) have been extensively studied for their role in promoting trained immunity. MPLA enhances glycolytic and oxidative capacities in murine and human leukocytes, providing protection against a broad spectrum of pathogens, including Gram-positive and Gram-negative bacteria and fungi, and conferring resistance against severe injury and sepsis.^9–16^ Collectively, these insights position trained innate immunity as a fundamental paradigm shift, broadening our understanding of innate immune function and its profound implications for human health and disease resilience.

The objective of this review is to explore the variability of trained innate immunity mechanisms and outcomes across different tissue microenvironments, with a focus on bone marrow, peripheral blood, and tissue-resident immune cells.

Collectively, it is well-established that TI is a highly complex biological process that necessitates the coordinated interplay of environmental sensing, hematopoietic activation, chromatin remodeling, and metabolic flux. These diverse factors collectively underscore the variability observed in TI's outcomes across different physiological and experimental contexts, each shaped by distinct training stimuli. Notably, evidence demonstrates that TI contributes to both beneficial and detrimental immune responses, including enhanced vaccine efficacy, efficient resolution of bacterial infections, chronic inflammation, atherosclerosis, and tumor immune surveillance.^4,11,13,17–23^ The current scientific challenge lies in deciphering how TI mechanisms operate across diverse tissue microenvironments. Specifically, understanding the similarities and distinctions of TI in bone marrow, peripheral blood, and tissue-resident immune cell compartments is crucial. Elucidating these mechanisms across various microenvironments will be pivotal for translating fundamental insights of TI into targeted therapeutic strategies, ultimately aiming to reduce morbidity and mortality associated with a wide spectrum of diseases.

## 2. Core molecular machinery of trained immunity

The hallmark of TI is a sustained, heightened responsiveness of innate immune cells that persists long after removal of the initial stimulus and occurs in the absence of antigen specificity. This phenomenon depends on stable reprogramming of myeloid cells and their progenitors through coordinated epigenetic, transcriptional, and metabolic changes.

### 2.1. Epigenetic reprogramming

The most consistent molecular signature of TI is the deposition of activating histone modifications, namely histone 3 lysine 4 trimethylation (H3K4me3) and histone 3 lysine 27 acetylation (H3K27ac), at promoters and enhancers of inflammatory and metabolic genes.^24,25^ ATAC-seq studies after BCG vaccination or β-glucan exposure show increased chromatin accessibility at these loci in HSPCs, monocytes, and macrophages. Critically, these marks are retained through myeloid differentiation and, in progenitors, can be transmitted to both myeloid and lymphoid lineages, explaining responses that outlast the lifespan of circulating monocytes.^3,5,25^ Loss-of-function experiments targeting writers (eg, SETD1, MLL1) or readers of these marks abolish trained phenotypes, confirming their functional necessity.^26,27^

While H3K4me3 and H3K27ac are extensively characterized as pivotal epigenetic markers of trained immunity (TI), the broader landscape of chromatin remodeling involved is far more complex. Among histone modifications, histone lactylation (the covalent addition of lactate to lysine residues on histones) has emerged as a novel activating mark that sustains inflammatory gene expression in trained macrophages. This modification directly links cellular metabolic states to chromatin accessibility through mechanisms distinct from classical methylation and acetylation pathways.^28,29^ Additionally, DNA methylation reprogramming contributes to the establishment and persistence of trained immune states: genome-wide bisulfite sequencing of BCG-trained monocytes revealed locus-specific hypomethylation at enhancers associated with cytokine and pattern recognition receptor genes.^30^ Pharmacological inhibition of DNA methyltransferases partially recapitulates transcriptional profiles observed in trained immunity.^31^ Furthermore, noncoding RNAs—including microRNAs, long noncoding RNAs, and circular RNAs—are increasingly recognized as critical post-transcriptional and epigenetic regulators that modulate inflammatory responses and chromatin-modifying enzymes following priming stimuli.^32^ Collectively, these interconnected layers of epigenetic regulation synergize with histone methylation and acetylation to shape the transcriptional programs underpinning trained innate immunity, underscoring the complexity and coordination of multiple mechanisms beyond a singular marker or pathway.

### 2.2. Linked metabolic and epigenetic circuits

**Figure 2:**
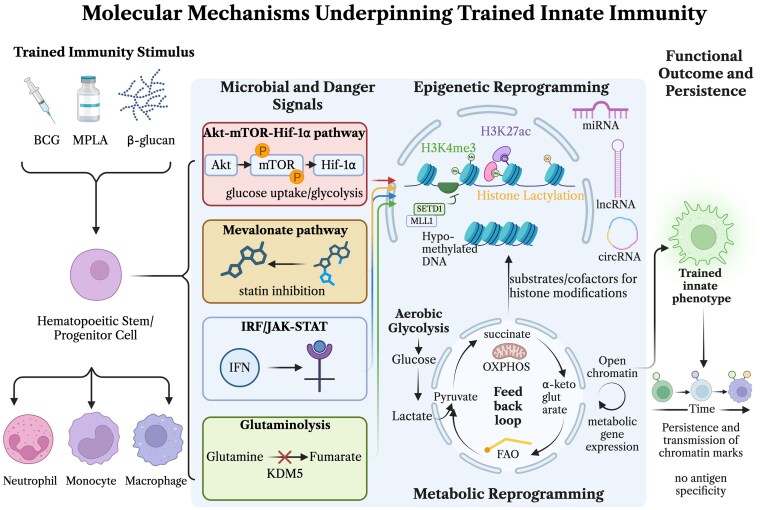
Molecular mechanisms underpinning trained innate immunity. Trained immunity stimuli, including BCG, MPLA, and β-glucan, act on hematopoietic stem and progenitor cells (HSPCs) and downstream myeloid lineage cells (neutrophils, monocytes, and macrophages) to induce long-lasting innate immune reprogramming. (Left) Upon encounter with microbial or danger signals, 4 major intracellular signaling axes are engaged: the Akt–mTOR–HIF-1α pathway, which promotes glucose uptake and glycolysis; the mevalonate pathway, which is inhibitable by statins; the IRF/JAK-STAT pathway, activated downstream of interferon (IFN) signaling; and glutaminolysis, wherein fumarate accumulation inhibits the histone demethylase KDM5. (Center) These pathways drive coordinated epigenetic reprogramming, including deposition of activating histone marks (H3K4me3, H3K27ac), histone lactylation, and DNA hypomethylation, mediated in part by chromatin-modifying complexes such as SETD1 and MLL1. Noncoding RNAs, including miRNAs, lncRNAs, and circRNAs, provide additional layers of epigenetic regulation. In parallel, metabolic reprogramming toward aerobic glycolysis generates key metabolic intermediates (succinate, α-ketoglutarate, lactate) that serve as substrates and cofactors for histone-modifying enzymes, establishing a feedback loop that reinforces open chromatin states and metabolic gene expression. Fatty acid oxidation (FAO) and oxidative phosphorylation (OXPHOS) also contribute to this metabolic circuitry. (Right) Together, these epigenetic and metabolic changes establish a trained innate phenotype characterized by persistent and heritable chromatin remodeling, enhanced responsiveness upon restimulation, and transmission of chromatin marks over time, all occurring in the absence of antigen specificity. Created with Biorender.com.

Epigenetic and metabolic reprogramming are inseparable pillars of trained immunity. The best-characterized model involves a shift toward aerobic glycolysis driven by Akt–mTOR–HIF-1α signaling, which sustains an inflammatory macrophage phenotype and deposits activating histone marks (H3K4me3, H3K27ac) at promoters of cytokines and metabolic genes.^24,33,34^ This creates a self-reinforcing loop: glycolytic intermediates serve as cofactors or substrates for histone-modifying enzymes, while open chromatin, in turn, amplifies the expression of glycolytic enzymes. However, this glycolytic prototype is not universal. Tissue-resident macrophages (TRMs) frequently operate under nutrient and oxygen constraints that favor oxidative phosphorylation (OXPHOS), fatty-acid oxidation (FAO), or glutaminolysis as baseline programs. Local metabolites (eg, short-chain fatty acids [SCFAs], succinate, α-ketoglutarate, or ischemia-derived lactate) can override or cooperate with the canonical mTOR–HIF-1α axis, leading to markedly different epigenetic and functional outcomes. Thus, the core molecular machinery provides a flexible scaffold that is profoundly reshaped by the tissue microenvironment (Fig. 2).

### 2.3. Key signaling nodes

Several conserved intracellular pathways integrate microbial and danger signals into the epigenetic-metabolic program described above. The Akt–mTOR–HIF-1α axis drives the canonical glycolytic shift in circulating monocytes,^33^ whereas the mevalonate pathway sustains membrane sterol synthesis and long-term training (a process abrogated by statins).^35^ Interferon-regulatory factor and JAK-STAT circuits amplify cytokine loops and myeloid-biased differentiation,^36^ and enhanced glutaminolysis leads to fumarate accumulation that inhibits KDM5 demethylases, thereby stabilizing H3K4me3 marks.^24^ Together, these signaling nodes form a shared molecular toolkit that is deployed differently depending on the anatomical niche and the nature of the priming stimulus.

## 3. Origins, transmission, and systemic spread of trained innate immunity

### 3.1. Hematopoietic origins and long-term transmission

The concept of TI fundamentally originates from and is sustained through dynamic modifications to hematopoietic stem cells (HSCs). HSCs express PRRs and directly respond to inflammatory cues,^37^ enabling them to undergo epigenetic and metabolic reprogramming that can be transmitted to their progeny.^38–40^ Despite their upstream position in the immune hierarchy, inflammatory stimuli reprogram HSCs in ways that shape downstream myeloid function, indicating that TI can be established at the level of hematopoiesis.^33,36,41^

Trained immunity is broadly conceptualized as operating through 2 complementary but mechanistically distinct programs: central trained immunity (CTM), in which HSPCs in the bone marrow are epigenetically reprogrammed and transmit trained phenotypes to their myeloid progeny, and peripheral trained immunity (PTM), in which fully differentiated innate immune cells (circulating monocytes and, critically, long-lived TRMs) are trained directly within their local tissue compartment. Although CTM was initially described as the dominant mechanism explaining long-term, nonspecific immune memory, PTM is increasingly recognized as an independent and tissue-autonomous process: TRMs can acquire and sustain trained states through local epigenetic reprogramming driven by tissue-specific cues, independently of renewed input from bone marrow progenitors.^42^ This distinction is foundational to the framework developed in this review, as the divergent functional outcomes observed across organs reflect not only the molecular toolkit imported via CTM-derived monocytes, but also the capacity of peripheral tissue macrophages to be trained and reprogrammed in situ by the microenvironmental signals unique to each niche.

Primary exposure to training agents such as BCG and β-glucan induces epigenetic modifications in HSCs, biasing differentiation toward the myeloid lineage while amplifying inflammatory potential.^3,5,43,44^ Transplantation and lineage-tracing studies demonstrate that trained phenotypes can persist well beyond the lifespan of circulating monocytes, supporting a progenitor-based mechanism of memory transmission.^3,5,43,44^ Yet, Kain et al.^41^ demonstrated that long-term HSC training with *Mycobacterium avium* did not suffice to maintain central TI, despite their self-renewal capacity.

Importantly, the durability of hematopoietic training appears to be stimulus dependent. Studies using broad PRR agonists such as BCG and β-glucan demonstrate robust and long-lasting epigenetic remodeling in HSCs, including sustained H3K4me3 deposition at inflammatory gene loci that can persist for years in humans.^3,5^ In contrast, stimuli engaging narrower PRR repertoires, such as *M. avium*, induce more transient training, suggesting that the strength and breadth of PRR signaling dictate whether reprogramming is enduring or short-lived.^41^

Differences in experimental design, timing of analysis, and progenitor populations examined further influence interpretations of HSC involvement. The interval between the initial stimulus and the observed training response also influences the outcomes. Some studies evaluate trained phenotypes within a short time frame of a few hours to days, while others assess over weeks to months, leading to disparities in results.^5,37,45^ Short-duration experiments may capture the presence of short-lived HSCs and activated myeloid cells; however, in longer models, cellular turnover suggests that only the most primitive HSC lineages can sustain a trained phenotype. These distinctions indicate that TI within the hematopoietic compartment exists along a spectrum rather than as a binary state.

Moreover, while TI predominantly involves innate immune cells, emerging evidence indicates that it also engages adaptive immunity, resulting in prolonged trained phenotypes even after the initial stimulus wanes. For instance, Lee et al. demonstrated that BCG vaccination confers long-term protection against SARS-CoV-2 via a positive feedback loop wherein CD4+ T cell-derived interferon-gamma (IFN-γ) induces an antiviral microenvironment in the lung, driven by activated IFN-γ-stimulated myeloid cells.^46^ This study highlights a complex interplay between innate and adaptive immune memory mechanisms. These collective discoveries also explain the durability of TI and the hematopoietic compartment as the central hub for TI-mediated inflammatory, metabolic, and epigenetic cues.

### 3.2. Peripheral blood monocytes: short-term effects and mobile carriers

Monocytes originate from HSCs. Circulating monocytes, along with other phagocytes of the innate immune system, serve as primary responders to infection and tissue damage. Under normal physiological conditions, the generation of myeloid cells from HSCs, as well as HSC self-renewal, is meticulously regulated to sustain daily blood cell production.^44,47–49^ Cytokines direct HSPCs to differentiate into either lymphoid or myeloid lineages. However, during acute circumstances such as injury, inflammation, and infection, hematopoiesis undergoes significant alterations, resulting in a predominance of phagocyte production—namely monocytes and granulocytes—while the production of lymphocytes and erythrocytes is deprioritized.^5^ TLR-mediated training promotes expansion of myeloid progenitors and mobilization of monocytes into circulation, positioning peripheral blood monocytes as immediate effectors and systemic carriers of trained immunity.^10^ Studies from other labs, like Li et al.^50^ also showed that TLR7/8 agonists expand bone marrow progenitors and increase the circulation of mature myeloid cells.

Circulating monocytes are not merely macrophage precursors but are functionally distinct, short-lived cells with high metabolic and epigenetic plasticity.^51^ Training stimuli such as BCG, β-glucan, and TLR agonists reprogram monocytes toward enhanced glycolysis via the conserved Akt–mTOR–HIF-1α axis, resulting in increased cytokine production, reactive oxygen species generation, and antimicrobial activity upon secondary challenge.^52–56^ These heightened responses are nonspecific to the original stimulus, reflecting deployment of a shared molecular toolkit rather than antigen-specific memory. Endogenous signals associated with metabolic diseases (including oxidized lipids, advanced glycation end products, and uric acid) can similarly induce trained phenotypes in circulating monocytes.^57^

### 3.3. Blood versus tissue reprogramming dynamics

There are key metabolic differences between circulating blood monocytes and TRMs. Blood monocytes are more metabolically flexible and are short-lived, capable of transitioning rapidly from resting to activated functional states upon stimuli. Upon tissue entry, local cues such as oxygen tension, nutrient availability, cytokines, and microbial metabolites recalibrate the trained program, often overriding the canonical glycolytic state observed in circulation. In contrast, TRMs rely predominantly on oxidative metabolism and require stronger or repeated inflammatory inputs to adopt trained characteristics.^58–62^

This mobility renders monocytes critical conduits linking systemic training to tissue-specific immunity but also introduces risk. In diseases associated with excessive or maladaptive inflammation, an increased monocyte inflammatory response to endogenous or exogenous stimuli can be deleterious. For example, during systemic tolerance induced by sepsis, reprogramming of innate immune cells leads to immune paralysis. In this context, innate immune cells exhibit increased apoptosis, impairments in antigen presentation, and reductions in antimicrobial functions such as ROS production, phagocytosis, and cytokine generation.^63,64^ Furthermore, while the heightened, long-lasting immune state conferred by training is highly advantageous in chronic inflammatory or autoimmune conditions induced by endogenous factors, it could paradoxically elevate disease risk. Evidence suggests that trained innate immunity can exacerbate disease pathology in conditions such as atherosclerosis, rheumatic diseases, and diabetes.^65–67^ Thus, peripheral blood monocytes exemplify how a conserved trained immunity toolkit can yield divergent outcomes once filtered through distinct tissue microenvironments.

## 4. Tissue-specific microenvironments shape trained innate immunity

**Figure 3:**
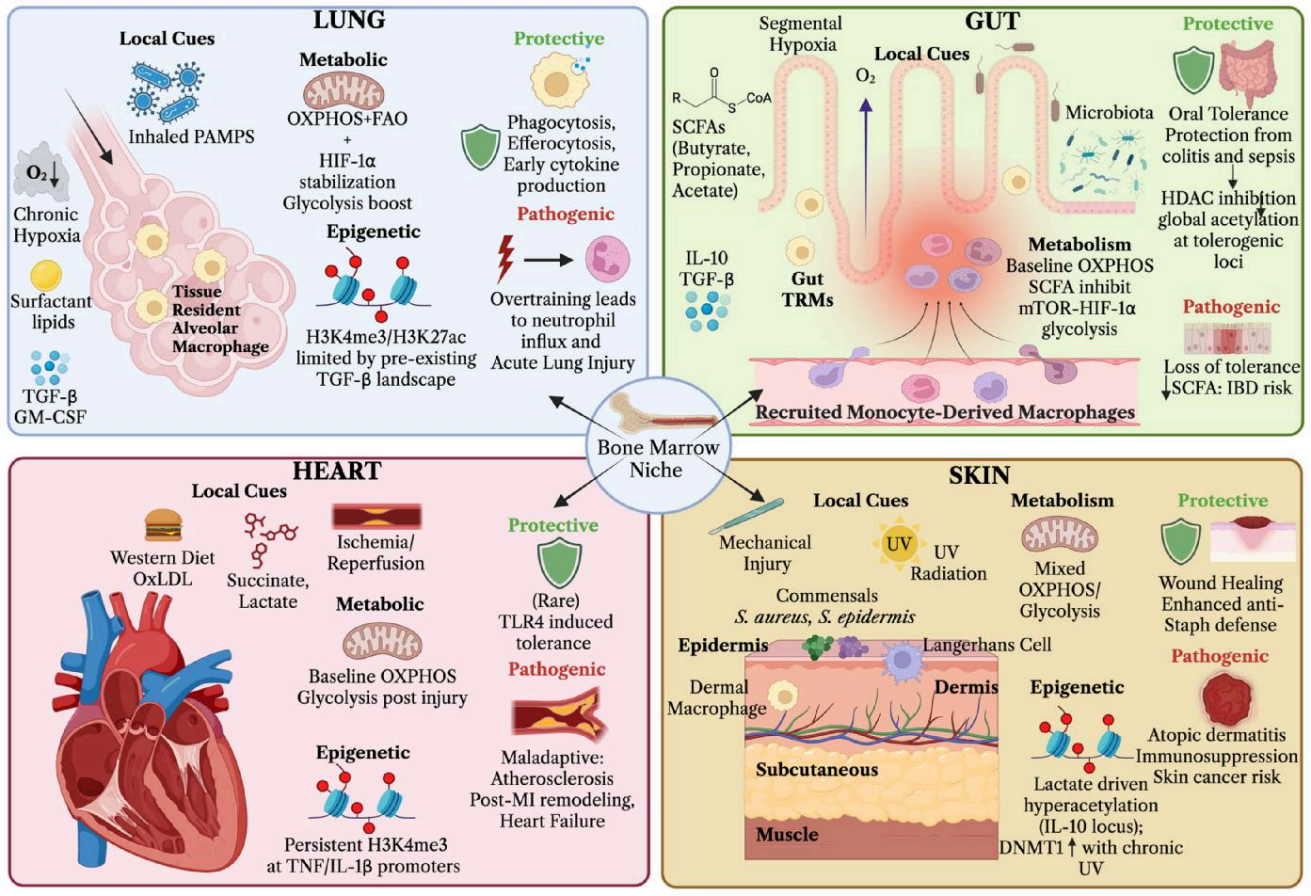
Trained innate immunity in tissue microenvironments. Tissue-specific microenvironments critically influence the induction and functional outcomes of trained immunity. Microenvironmental cues modulate the magnitude and nature of TI responses across different tissues. In the pulmonary tissue, exposure to inhaled pathogens or canonical training agents such as LPS and β-glucan induces metabolic rewiring characterized by enhanced oxidative metabolism and epigenetic modifications, notably the deposition of H3K4me3 and H3K27ac marks at gene promoters and regulatory regions. These epigenetic changes potentiate alveolar macrophage antimicrobial capacity and facilitate monocyte recruitment, thereby expediting pathogen eradication. Nonetheless, excessive stimulation may precipitate hyperinflammation, tissue injury, and acute lung pathology. In the intestinal milieu, ongoing contact with commensal microbiota and dietary antigens promotes a metabolic program driven by short-chain fatty acids (SCFAs). Concurrently, reprogramming of IL-10 and TGF-β pathways fosters tolerogenic mechanisms in tissue-resident monocytes and macrophages. TI-associated metabolic shifts involve mTOR-HIF1α-dependent glycolytic enhancement; however, precise regulation of this signaling axis is essential to prevent excessive inflammatory responses. In the cardiac tissue, where microbial exposure is minimal under physiological conditions, stimuli such as ischemia, oxidized LDL, or a Western diet induce maladaptive training. Epigenetic reprogramming at IL-1β and TNFα loci facilitates foam cell formation and accelerates atherogenesis. In the skin, constant exposure to commensals and environmental insults like UV radiation establishes baseline training for Langerhans cells and dermal macrophages. These resident immune cells rapidly secrete antimicrobial peptides to defend against pathogens; however, dysregulation or hypertraining may lead to chronic inflammatory states. Overall, trained immunity represents a dynamic process capable of conferring protection, promoting repair, or driving pathology depending on tissue context and stimulus intensity. Created with Biorender.com.

TRMs also play a pivotal role in the immune response, comparable to that of circulating monocytes. The functional reprogramming following trained innate immunity and its ultimate effects on antimicrobial and inflammatory responses are predominantly modulated by tissue-specific cues and local environmental stimuli. The microenvironment of organs such as the lung, gut, heart, and skin exhibits significant heterogeneity, which can influence macrophage functionality. Key tissue-specific factors include microbiota composition, oxygen tension, and exposure to exogenous PAMPs, all of which can modulate macrophage training and activity (Fig. 3).

While the training of progenitors and peripheral cells is foundational to TI and its enhanced response, these signals ultimately converge in the tissues where long-lived tissue-resident cells are reprogrammed and orchestrate TI locally. Tissue-specific environments profoundly influence TI, with local cues modulating both the character and magnitude of immune responses. For instance, TRMs in the lung, gut, heart, and skin can be differentially programmed in response to variations in oxygen tension, cytokine signaling, and basal transcriptional and metabolic programs tailored by the tissue microenvironment.^68^ These factors result in distinct responses of TRMs to stimuli compared with those of circulating monocytes, enabling tissue-adapted immune functions. Notably, microbiota in the gut and skin significantly impact the epigenetic landscape of resident immune cells, promoting immune tolerance in environments where contact with foreign stimuli is frequent.^69^ This tolerance is essential for preventing autoimmunity and excessive immune responses that could lead to cytokine storms and tissue damage. Additionally, hypoxia shapes immune outcomes in tissues such as the lung, where glycolytic reprogramming is often predominant. Hypoxic tissues utilize HIF-1α stabilization to drive glycolysis and energy production under low oxygen conditions.^70^ Conversely, nutrient-poor environments, such as the heart, rely predominantly on OXPHOS, which maintains energy homeostasis but may constrain overall metabolic flux between OXPHOS and glycolysis. These metabolic adaptations are critical for tailored immune responses within distinct tissue microenvironments.

### 4.1. Lung: hypoxia and inhaled stimuli

The adult lung harbors 2 major tissue-resident macrophage populations, alveolar macrophages (AMs) in the airway lumen and interstitial macrophages in the parenchyma, that are predominantly of embryonic origin and self-maintain with minimal monocyte contribution.^8,71^ The pulmonary microenvironment is profoundly hypoxic (alveolar PO_2_ ∼ 35-40 mmHg), glucose-poor, and rich in TGFβ and GM-CSF, as well as in surfactant lipids.^71^ These cues enforce a quiescent, tolerogenic phenotype characterized by reliance on OXPHOS and FAO, active suppression of inflammation, and high efferocytic capacity (Table 1).

**Table 1:** Tissue specific trained innate immunity.

Tissue	Dominant local cues	Baseline metabolism of TRMs	Effect on canonical TI program (Section 2)	Epigenetic bias	Protective outcome	Pathologic outcome
Lung	Chronic hypoxia, inhaled PAMPs, surfactants	OXPHOS + FAO	Amplifies via mTOR-independent HIF-1α; requires glutaminolysis/FAO	H3K4me3/H3K27ac limited by TGF-β landscape	Rapid bacterial/viral clearance, enhanced efferocytosis	Acute lung injury from over-training (eg, systemic β-glucan)
Gut	SCFAs, IL-10, TGF-β, segmental hypoxia	OXPHOS	Strongly suppresses mTOR–HIF-1α–glycolysis; HDAC inhibition dominates	Global hyperacetylation at tolerogenic loci	Oral tolerance, protection from colitis & sepsis	Loss of tolerance → IBD when SCFA brake is removed
Heart	Ischemia → succinate/lactate, oxLDL, Western diet	OXPHOS (steady-state) → glycolysis postinjury	Potentiates and sustains canonical program; few endogenous brakes	Persistent H3K4me3 at TNF/IL-1β promoters	Rare (TLR4-induced tolerance possible)	Accelerated atherosclerosis, post-MI remodeling, heart failure
Skin	Commensal S. epidermidis (lactate, LTA), UV, mechanical injury	Mixed OXPHOS/glycolysis	Commensals inhibit HDAC11 → tolerance; UV bimodal (protective vs immunosuppressive)	Lactate-driven hyperacetylation; DNMT1↑ with chronic UV	Wound healing, anti-staphylococcal defense	Atopic dermatitis, immunosuppression → skin cancer risk

In striking contrast to circulating monocytes, which adopt the canonical Akt–mTOR–HIF-1α–glycolysis circuit upon training, lung TRMs operate on a metabolic baseline that restrains this pathway. Nevertheless, inhaled microbial products such as low-dose LPS or β-glucan can induce robust TI in AMs, revealing a highly adapted version of the universal program.^71–75^ Chronic hypoxia stabilizes HIF-1α independently of mTOR, allowing secondary inflammatory stimuli to trigger rapid glycolytic boosting without the full canonical upstream signaling cascade.^75^ Moreover, lung TRMs require metabolic flexibility: blocking either glutaminolysis or FAO completely abolishes the trained phenotype, whereas glycolysis inhibition alone has only partial effects.^75^ Training still deposits the expected H3K4me3 and H3K27ac marks at inflammatory gene promoters, but the pre-existing TGF-β- and surfactant-driven chromatin landscape limits the magnitude and duration of the response, preventing uncontrolled inflammation.^75,76^

These adaptations confer clear protective benefits. Low-dose inhaled TLR agonists (eg, the novel TLR4 ligand 3D-PHAD) or bacterial priming enhance resistance to subsequent *Klebsiella pneumoniae* or Pseudomonas challenge by increasing phagocytic uptake, efferocytosis, and early cytokine production.^77,78^ Trained AMs up-regulate Mertk, Marco, and CD163, promoting resolution and repair.^78^ However, the same hypoxic, metabolically poised environment makes the lung exquisitely sensitive to over-training. Systemic β-glucan, which strongly engages the canonical glycolytic program in circulating cells, reprograms AMs maladaptively: upon secondary LPS exposure, these cells drive exaggerated neutrophil influx and acute lung injury marked by elevated CXCL1, TNF-α, and IL-6 levels.^79^

Notably, a noncanonical form of TI operates in AMs that is independent of the classical Akt–mTOR–HIF-1α–glycolysis axis: intranasal LPS exposure trains resident AMs through a pathway requiring type I interferon signaling, with FAO and glutaminolysis, rather than glycolysis, serving as the obligate metabolic substrates,^29^ revealing that the identity of the initiating signal, not just its presence, determines which arm of the TI toolkit is engaged.^70^

Thus, in the lung, the universal TI toolkit is transformed into a hypoxia-tuned, metabolically flexible, and tightly regulated response: highly protective when appropriately calibrated, yet uniquely prone to tissue-damaging hyperinflammation when the glycolytic circuit is forced beyond physiological limits. Timing, dose, route, and pre-existing oxygen tension are the critical determinants of whether training manifests as rapid pathogen containment or detrimental acute lung injury (Table 1).

### 4.2. Gut: microbiota-derived metabolites and tolerance

Gut tissue-resident TRMs in the lamina propria are continuously replenished from circulating monocytes and must maintain strict hyporesponsivity to trillions of commensal microbes and food antigens while remaining poised to eliminate pathogens.^80–82^ This tolerogenic phenotype is enforced by an array of local signals (IL-10, TGF-β), and especially SCFAs (butyrate, propionate, acetate), that collectively drive a baseline program of OXPHOS and actively restrain the proinflammatory circuitry that dominates TI in circulating monocytes^83–85^ (Table 1).

In sharp contrast to the canonical Akt-mTOR-HIF-1α-glycolysis axis described above, gut TRMs experience profound inhibition of this pathway under steady-state conditions. SCFAs inhibit mTOR signaling, suppress HIF-1α stabilization, and shift metabolism away from glycolysis toward oxidative programs. At the epigenetic level, butyrate and propionate act as potent HDAC inhibitors, increasing global histone acetylation and altering chromatin accessibility at anti-inflammatory loci.^84–86^ The net result is reduced IL-6 and IL-12 production upon stimulation, with preserved or even enhanced TNF-α responses that support controlled antimicrobial defense without collateral tissue damage.^84,87^ This constitutes a noncanonical TI scenario: rather than activating the mTOR–HIF-1α–glycolysis circuit, microbiota-derived butyrate functions as an HDAC inhibitor in intestinal macrophages, increasing histone acetylation at secondary response gene promoters while paradoxically suppressing proinflammatory cytokine output,^88^ a tolerogenic epigenetic memory state that is mechanistically inverted relative to classical β-glucan or BCG-induced training.^79^

This tolerogenic metabolic and epigenetic baseline has direct consequences for TI. When classical training stimuli such as β-glucan or BCG are encountered in the gut microenvironment, the SCFA-rich niche blunts the expected glycolytic burst and limits deposition of strong H3K4me3/H3K27ac marks at proinflammatory gene promoters. Instead, training is redirected toward enhanced antimicrobial readiness within a tolerogenic framework. Oral β-glucan, for example, reprograms intestinal macrophages to confer protection against both subsequent colitis and systemic bacterial challenge, demonstrating that gut-specific training can exert beneficial effects locally and at distant sites.^7,89^

Spatial heterogeneity within the gut further refines this process. Crypt macrophages reside in a more hypoxic niche and exhibit constitutive HIF-1α stabilization that partially mimics a “pseudo-trained” state, whereas villus-tip macrophages near oxygenated capillaries rely predominantly on OXPHOS. Amino acid metabolites such as tryptophan-derived kynurenine reinforce anti-inflammatory histone modifications and suppress TNF-α, IL-17, and IL-23 release,^90–93^ while glutamine-driven fumarate accumulation can still inhibit KDM5 demethylases and sustain some activating marks.^24^ Thus, even within the same organ, the balance between proinflammatory and tolerogenic trained phenotypes varies along the crypt-villus axis.

Systemic training stimuli (eg, intraperitoneal β-glucan or BCG) can also reach the gut and induce epigenetic changes in resident macrophages, but the final functional outcome is invariably filtered through the SCFA-dominated niche.^45^ Consequently, gut TRMs rarely adopt the full proinflammatory trained phenotype seen in blood monocytes or lung AMs. Overriding this tolerogenic brake, through dysbiosis, loss of SCFA production, or excessive systemic training, risks breakdown of oral tolerance and the development of inflammatory bowel disease.

In summary, the gut microenvironment actively antagonizes the canonical TI program, replacing robust glycolysis and proinflammatory epigenetics with oxidative metabolism and tolerance-promoting chromatin modifications. This adaptation allows gut macrophages to integrate microbial training signals without precipitating chronic inflammation, illustrating how the same molecular toolkit can be repurposed for diametrically opposite functional outcomes depending on the tissue niche (Table 1).

### 4.3. Heart: ischemia, western diet, and maladaptive training

The adult heart contains a large, self-renewing population of embryonic-derived TRMs that coexist with monocyte-derived cells, especially after injury. Under steady-state conditions, cardiac macrophages rely predominantly on OXPHOS and FAO, reflecting the heart's high energetic demand and constant oxygen supply. However, 2 dominant insults—ischemia/reperfusion and chronic exposure to a Western diet or oxidized low-density lipoprotein (oxLDL)—systematically divert the universal TI program toward exaggerated and often pathologic inflammation (Table 1).

Ischemic injury rapidly stabilizes HIF-1α, even after reperfusion, and triggers accumulation of succinate and lactate, oncometabolites that sustain IL-1β production via HIF-1α-dependent mechanisms.^94–96^ This ischemia-driven program represents a noncanonical TI scenario in which the training stimulus is entirely endogenous, succinate and lactate accumulating from tissue necrosis rather than exogenous PAMPs, with TCA cycle intermediates such as succinate directly altering epigenetic regulation of proinflammatory cytokine genes in cardiac macrophages through mechanisms that mirror, but are biochemically distinct from, classical β-glucan-induced H3K4me3 deposition.^32^ These metabolic shifts mimic and amplify the canonical glycolytic training signature, depositing H3K4me3 and H3K27ac marks at inflammatory gene promoters in both resident and recruited macrophages. The result is a maladaptive “ischemic memory” that heightens responsiveness to subsequent insults, promoting excessive neutrophil recruitment, adverse ventricular remodeling, and progression to heart failure.^97,98^

Western diet and oxLDL exert an equally potent, but more chronic, training effect. Hypercholesterolemia reprograms bone marrow progenitors through NLRP3 inflammasome activation and sustained mevalonate pathway flux, biasing myelopoiesis toward inflammatory Ly6C^hi^ monocytes.^99–103^ These cells seed the heart and vasculature, where oxLDL further engages LOX-1 and CD36, driving foam-cell formation and sustained cytokine release.^35,104^ Alternating high-fat diet regimens accelerate atherosclerosis even in lymphocyte-deficient Rag2^−/−^ mice, confirming that myeloid-TI is the primary driver.^102^ Epigenetic analyses reveal persistent H3K4me3 enrichment at promoters of TNF-α, IL-1β, and IL-6 in cardiac macrophages from hypercholesterolemic animals, establishing a feed-forward loop that exacerbates plaque instability and postinfarct inflammation.^103^

Protective TI in the heart is possible but rare. Low-dose TLR4 agonists can induce tolerance rather than training, reducing inflammation and preserving function after ischemic or metabolic stress through enhanced IFN signaling and myeloid cross-talk.^105^ Exercise and caloric restriction reverse maladaptive progenitor reprogramming, highlighting the heart's sensitivity to systemic metabolic cues.^106–108^

Thus, unlike the lung (where hypoxia restrains over-training) or the gut (where SCFAs enforce tolerance), the cardiac microenvironment lacks strong endogenous brakes on the canonical proinflammatory program. Ischemia and the Western diet instead act as persistent endogenous training stimuli that convert the universal molecular toolkit into a driver of chronic cardiovascular disease. This maladaptive bias explains why TI in the heart is predominantly pathologic and why interventions that interrupt NLRP3, mevalonate, or oxLDL signaling hold particular therapeutic promise.

### 4.4. Skin: UV radiation, commensals, and barrier surveillance

The skin maintains 2 distinct macrophage compartments: the epidermal Langerhans cells and dermal macrophages. Together, these form the body's most exposed innate immune sentinel network. Unlike the lung or heart, skin macrophages are constantly exposed to microbial colonization, frequent mechanical or chemical injury, and cyclic UV radiation, creating a microenvironment that simultaneously promotes and restrains TI.

Commensal *Staphylococcus epidermidis* is the dominant physiologic trainer of skin myeloid cells. Through lipoteichoic acid-TLR2 engagement and lactate production, *S. epidermidis* inhibits HDAC11, drives histone hyperacetylation (especially at the IL-10 locus), and primes dermal macrophages and Langerhans cells for enhanced IL-17 and antimicrobial peptide responses upon secondary challenge.^109–114^ This commensal-induced program deviates sharply from the canonical mTOR–HIF-1α–glycolysis axis: it favors mixed oxidative/glycolytic metabolism and enforces an anti-inflammatory, wound-healing phenotype that accelerates barrier restoration without tissue-damaging hyperinflammation.^112,113,115^ This represents a noncanonical TI pathway in which the training signal is commensal-derived lactate rather than a classical PAMP: bacterial lactate directly inhibits HDAC11, driving histone hyperacetylation and sustained IL-10 expression^116^ in a manner that diverges sharply from the canonical mTOR–glycolysis circuit and instead exploits a metabolite-sensor axis unique to the cutaneous microenvironment.

In contrast, repeated or high-burden exposure to *Staphylococcus aureus* flips the same molecular toolkit toward maladaptive training. *S. aureus* proteases disrupt barrier integrity, and its glycolytic metabolism limits fumarate production, impairing KDM5 inhibition and thus reducing stable TI.^117^ Persistent or dysbiotic *S. aureus* instead drives exaggerated TNF-α, IL-6, and IL-17 responses via sustained histone acetylation and open chromatin at proinflammatory loci, contributing to chronic conditions such as atopic dermatitis and Netherton syndrome.^118^

UV radiation adds another layer of complexity. Acute UVB activates PRRs through DNA damage and induces clonal expansion of hematopoietic progenitors, while chronically elevating DNA methyltransferase-1 (DNMT1) expression.^119,120^ The net effect is bimodal: moderate UV primes protective training responses (reduced multiple sclerosis risk via vitamin D-independent pathways^121^), whereas prolonged or high-dose exposure triggers immunosuppressive training, increasing skin cancer susceptibility through poorly characterized epigenetic silencing of antitumor surveillance genes.

Epidermal stem cells themselves acquire inflammatory memory. Following imiquimod- or wound-induced inflammation, specific chromatin domains remain accessible, enabling faster regeneration and cytokine production upon reinjury.^122–124^ Recruited dermal macrophages integrate these stem-cell signals with progenitor-level training sustained by GM-CSF, ensuring that even high-turnover myeloid populations retain functional memory.^125^

Thus, the skin repurposes the universal TI machinery into a uniquely bidirectional program: commensal-driven, HDAC11/lactate-dependent tolerance that promotes healing and barrier maintenance, versus pathogen- or UV-driven maladaptive training that risks chronic inflammation or immunosuppression. This duality reflects the skin's evolutionary mandate to tolerate a heavy microbial burden while remaining poised against invasive threats and neoplastic transformation (Table 1).

## 5. Clinical translation and therapeutic opportunities

The tissue-specific deviations from the canonical TI program described above define both the promise and the peril of therapeutic intervention. The same molecular machinery that protects the lung against pneumonia or maintains gut tolerance can drive atherosclerosis in the heart and immunosuppression in chronically UV-exposed skin. Successful translation, therefore, requires precise spatial control, inducing or blocking TI only in the intended niche, rather than the blunt systemic approaches that dominated early clinical studies.

Intravesical BCG remains the most mature example of tissue-restricted TI. By acting locally on urothelial and bladder-resident macrophages, BCG sustains epigenetic reprogramming and high IL-6/TNF-α responses, thereby preventing recurrence of nonmuscle-invasive bladder cancer in 60% to 70% of patients.^21,126^ Ongoing phase III trials combining BCG with IL-15 superagonist N-803 (QUILT-3.032) or checkpoint inhibitors exploit this localized training to achieve complete response rates exceeding 70% in BCG-unresponsive disease. In the respiratory tract, inhaled β-glucan is entering phase I/II trials, leveraging the lung's hypoxia-tuned circuitry to confer short-term protection against viral and bacterial pneumonia in high-risk elderly populations (ACTIVATE trial, NCT03619252) without the maladaptive cardiac effects seen with systemic administration.

Conversely, maladaptive training is already being targeted pharmacologically. Statins, colchicine, and bempedoic acid interrupt the mevalonate-NLRP3 axis that sustains Western-diet-driven cardiac reprogramming^103,127–129,130^; colchicine reduced secondary cardiovascular events by 23% in the CANTOs and COLCOT trials by preventing persistent H3K4me3 deposition in cardiac macrophages. In sepsis-induced immunoparalysis, recombinant IL-4-Fc fusion proteins that selectively re-tolerize myeloid cells are advancing through preclinical testing, restoring oxidative and glycolytic metabolism in exhausted monocytes. Revelation Biosciences' Gemini—a proprietary intravenous formulation of phosphorylated hexaacyl disaccharide (PHAD^®^), a TLR4 agonist—is in phase 1b development (PRIME study) for chronic kidney disease, following positive phase 1 safety data showing biomarker activity consistent with innate immune rebalancing; preclinical models^131^ support its potential to prevent acute kidney injury in ischemic settings by preconditioning monocytes prior to tissue seeding.

Emerging delivery platforms now make true tissue specificity feasible. Inhaled or nanoparticle-encased TLR agonists confine training to the lung,^132–135^ gut-restricted HDAC activators or SCFA prodrugs reinforce tolerogenic programming in inflammatory bowel disease,^136^ and topical TLR7/8 agonists combined with vitamin D analogs are being developed to drive protective cutaneous training in chronic wounds or high-risk skin cancer patients.^137,138^ Single-cell ATAC-seq and spatial transcriptomics provide companion diagnostics,^139,140^ identifying lung-specific IRF7 enhancers that mark beneficial training versus cardiac NLRP3-driven enhancers that signal pathology.^141^ Organoid–immune co-culture models of lung, gut, heart, and skin now enable rapid screening of these niche-restricted inducers before expensive animal studies.^142–144^

**Figure 4:**
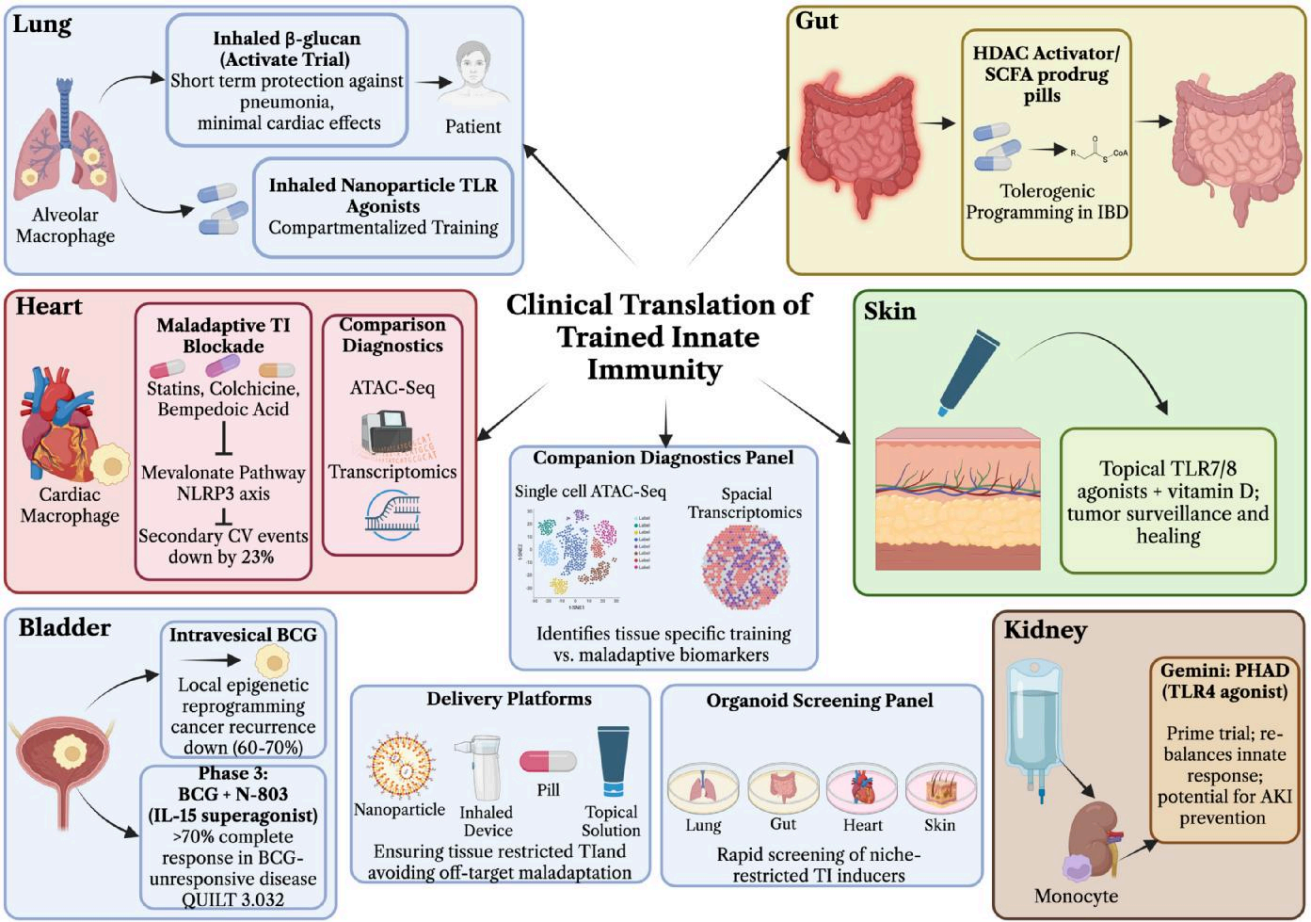
Clinical translation of trained immunity. An overview of recent clinical advancements exploiting tissue-specific modulation of trained immunity. Evidence indicates that BCG therapy elicits localized immune responses within the bladder, characterized by increased IL-6 and TNF-alpha cytokine production alongside epigenetic reprogramming of innate immune cells. Combination strategies incorporating IL-15 have demonstrated enhanced efficacy, achieving complete response rates in patients unresponsive to BCG alone. In pulmonary applications, inhaled B-glucan has conferred transient protection against pneumonia with limited systemic dissemination, while nanoparticle-based Toll-like receptor (TLR) agonists enable highly targeted immune training within specific compartments. Cardiac interventions aim to inhibit maladaptive immune activation through agents such as statins, colchicine, and bempedoic acid by suppressing the mevalonate pathway and NLRP3 inflammasome activation, thereby reducing adverse cardiovascular events. In gastrointestinal tissues, the use of HDAC activators and short-chain fatty acid (SCFA) analogs promotes tolerance mechanisms pertinent to inflammatory bowel disease management. Melanoma and other skin cancers show responsiveness to topical TLR antagonists combined with vitamin D, leading to improved outcomes. The integration of cutting-edge diagnostic platforms, including single-cell RNA sequencing, ATAC-seq, spatial transcriptomics, and organoid models, facilitates precise, tissue-specific induction of beneficial trained immunity while minimizing off-target or detrimental effects. Ultimately, clinical translation hinges on sophisticated targeting and delivery systems that enable personalized, tissue-resident modulation of innate immune memory, advancing therapeutic precision. Created with Biorender.com.

The clinical future of TI therefore lies in exploiting, rather than overriding, the tissue-specific adaptations outlined in this review. Therapeutic success will hinge on delivery vehicles and biomarkers that restrict reprogramming to the desired compartment—lung for infection protection, gut for tolerance restoration, heart for atherosclerosis prevention, and skin for wound healing or tumor surveillance—while preventing off-target maladaptation elsewhere^145^ (Fig. 4).

## 6. Unresolved questions and future directions

Despite rapid progress, the tissue-centric framework presented here exposes several critical gaps that now define the frontier of TI research. First, we lack a quantitative understanding of how systemic training stimuli, whether canonical (BCG, β-glucan, TLR agonists) or noncanonical (Western diet, ischemia, UV exposure), are filtered, amplified, or silenced as circulating monocytes enter distinct niches. Does the lung's hypoxia-tuned circuitry simply accelerate decay of systemic marks, or does it actively overwrite them with local epigenetic signatures? Conversely, why does the cardiac microenvironment appear uniquely permissive, allowing even modest systemic oxLDL or succinate signals to establish decades-long maladaptive memory?

Second, the persistence of trained phenotypes across turnover rates remains poorly mapped. Lung AMs are embryonically derived and extraordinarily long-lived. Yet, we do not know whether their training is intrinsically more durable than the short-lived, monocyte-replenished populations of the gut lamina propria. Direct head-to-head comparisons, using barcoded HSPCs or fate-mapping tools, are now needed to determine whether epigenetic memory is primarily cell-intrinsic or niche-sustained.

Third, we have almost no data on the decay kinetics of tissue-specific marks. Do H3K4me3/H3K27ac landscapes in cardiac macrophages erode predictably after statin therapy, or does the ischemic niche re-impose them? Can gut SCFA levels be therapeutically titrated to accelerate loss of maladaptive training in IBD? Answering these questions requires longitudinal, multi-organ single-cell ATAC-seq and spatial metabolomics in the same animals or patients before and after intervention.

Finally, the most translationally urgent gap is the absence of biomarkers that distinguish beneficial from pathological training within a given tissue. Lung-specific IRF7-driven enhancers, gut SCFA-responsive HDAC targets, cardiac NLRP3-dependent loops, and skin lactate/HDAC11 signatures all represent candidate “training barcodes” that could guide patient stratification and monitor on-target effects of emerging therapies.

The tools to close these gaps—tissue-specific organoids co-cultured with barcoded trained monocytes, spatial multi-omics, and reversible CRISPR epigenetic editors—are already available. Addressing them will transform TI from a fascinating biological phenomenon into a clinically actionable axis, enabling therapies that induce protective memory exactly where it is needed (the lung, gut, skin) while preventing maladaptive memory where it is harmful (the heart, chronically inflamed barrier sites).

While the framework presented in this review centers on myeloid immune cells and their progenitors, growing evidence suggests that TI is not exclusive to the immune system. Nonimmune cells such as epithelial cells, fibroblasts, and stromal and epidermal stem cells possess memory-like characteristics consistent with TI.^29,146^ Most compellingly for the tissue-centric argument developed here, skin epithelial stem cells retain a prolonged memory of acute inflammation that enables them to hasten barrier restoration after subsequent tissue damage, maintaining chromosomal accessibility at key stress response genes activated during the primary stimulus.^124^ Vascular smooth muscle cells exposed to oxLDL or BCG similarly adopt a sustained proinflammatory phenotype via mTOR-HIF1α signaling, increased aerobic glycolysis, and epigenetic reprogramming of inflammatory gene promoters,^116^ mechanistically mirroring what has been characterized in trained monocytes. These findings raise important speculative questions for the tissue-specific model presented here: if structural cells like epithelial, endothelial, and fibroblastic cells within a given niche can themselves be trained, they may amplify, sustain, or even initiate the local epigenetic and metabolic signals that shape macrophage training in that same compartment. In the gut, for instance, trained intestinal epithelial progenitors could reinforce the tolerogenic baseline that restrains macrophage inflammatory responses, while in the skin, inflammation-experienced epithelial stem cells and trained dermal fibroblasts may cooperate with Langerhans cells and dermal macrophages to coordinate barrier repair. Conversely, in the heart, trained smooth muscle cells and endothelial cells primed by oxLDL or ischemia may compound the maladaptive training of cardiac macrophages, creating a feed-forward inflammatory loop that accelerates atherosclerosis beyond what macrophage training alone can explain. Defining the relative contributions of immune and nonimmune cell training within each tissue niche represents a critical open question for the field, and one with direct therapeutic implications. The tissue-specific lens introduced in this review provides the conceptual roadmap; the next decade of experimental work will determine whether we can navigate it with precision.

## Data Availability

Data will be made available upon reasonable request.

## References

[1] Netea MG, Quintin J, van der Meer JWM. Trained immunity: a memory for innate host defense. Cell Host Microbe. 2011:9:355–361. 10.1016/j.chom.2011.04.006 21575907

[2] Di Luzio NR, Williams DL. Protective effect of glucan against systemic Staphylococcus aureus septicemia in normal and leukemic mice. Infect Immun. 1978:20:804–810. 10.1128/iai.20.3.804-810.1978 352959 PMC421929

[3] Kaufmann E et al BCG educates hematopoietic stem cells to generate protective innate immunity against Tuberculosis. Cell. 2018:172:176–190.e19. 10.1016/j.cell.2017.12.031 29328912

[4] Redelman-Sidi G, Glickman MS, Bochner BH. The mechanism of action of BCG therapy for bladder cancer—a current perspective. Nat Rev Urol. 2014:11:153–162. 10.1038/nrurol.2014.15 24492433

[5] Mitroulis I et al Modulation of myelopoiesis progenitors is an integral component of trained immunity. Cell. 2018:172:147–161.e12. 10.1016/j.cell.2017.11.034 29328910 PMC5766828

[6] Walk J et al Controlled human malaria infection induces long-term functional changes in monocytes. Front Mol Biosci. 2020:7:604553. 10.3389/fmolb.2020.604553 33324683 PMC7726436

[7] Stothers CL et al β-Glucan induces distinct and protective innate immune memory in differentiated macrophages. J Immunol (Baltimore, Md.: 1950). 2021:207:2785–2798. 10.4049/jimmunol.2100107 PMC8612974 34740960

[8] Kleinnijenhuis J et al Bacille Calmette-Guerin induces NOD2-dependent nonspecific protection from reinfection via epigenetic reprogramming of monocytes. Proc Natl Acad Sci U S A. 2012:109:17537–17542. 10.1073/pnas.1202870109 22988082 PMC3491454

[9] Hernandez A et al The role of MyD88- and TRIF-dependent signaling in monophosphoryl lipid A-induced expansion and recruitment of innate immunocytes. J Leukoc Biol. 2016:100:1311–1322. 10.1189/jlb.1A0216-072R 27354411 PMC5109999

[10] Bohannon JK et al Role of G-CSF in monophosphoryl lipid A-mediated augmentation of neutrophil functions after burn injury. J Leukoc Biol. 2016:99:629–640. 10.1189/jlb.4A0815-362R 26538529 PMC4787290

[11] Fensterheim BA et al The TLR4 agonist monophosphoryl lipid A drives broad resistance to infection via dynamic reprogramming of macrophage metabolism. J Immunol (Baltimore, Md.: 1950). 2018:200:3777–3789. 10.4049/jimmunol.1800085 PMC5964009 29686054

[12] Fukuda S et al Monophosphoryl lipid a attenuates multiorgan dysfunction during post-burn *Pseudomonas aeruginosa* pneumonia in sheep. Shock (Augusta, Ga.). 2020:53:307–316. 10.1097/SHK.0000000000001364 31045990 PMC6937402

[13] McBride MA et al Bacteria- and fungus-derived PAMPs induce innate immune memory via similar functional, metabolic, and transcriptional adaptations. J Leukoc Biol. 2024:115:358–373. 10.1093/jleuko/qiad120 37793181 PMC10872320

[14] Owen AM et al MyD88-dependent signaling drives toll-like receptor-induced trained immunity in macrophages. Front Immunol. 2022:13:1044662. 10.3389/fimmu.2022.1044662 36439136 PMC9692127

[15] Romero CD et al The toll-like receptor 4 agonist monophosphoryl lipid a augments innate host resistance to systemic bacterial infection. Infect Immun. 2011:79:3576–3587. 10.1128/IAI.00022-11 21646453 PMC3165493

[16] Watts BA, Tamayo E, Sherwood ER, Good DW. Monophosphoryl lipid A pretreatment suppresses sepsis- and LPS-induced proinflammatory cytokine production in the medullary thick ascending limb. Am J Physiol Renal Physiol. 2020:319:F8–F18. 10.1152/ajprenal.00178.2020 32421349 PMC7468828

[17] Dagenais A, Villalba-Guerrero C, Olivier M. Trained immunity: a “new” weapon in the fight against infectious diseases. Front Immunol. 2023:14:1147476. 10.3389/fimmu.2023.1147476 36993966 PMC10040606

[18] Aaby P, Benn CS. Saving lives by training innate immunity with Bacille Calmette-Guerin vaccine. Proc Natl Acad Sci U S A. 2012:109:17317–17318. 10.1073/pnas.1215761109 23071307 PMC3491466

[19] Covián C, Retamal-Díaz A, Bueno SM, Kalergis AM. Could BCG vaccination induce protective trained immunity for SARS-CoV-2? Front Immunol. 2020:11:970. 10.3389/fimmu.2020.00970 32574258 PMC7227382

[20] Netea MG et al Trained immunity: a tool for reducing susceptibility to and the severity of SARS-CoV-2 infection. Cell. 2020:181:969–977. 10.1016/j.cell.2020.04.042 32437659 PMC7196902

[21] Van Puffelen JH et al Trained immunity as a molecular mechanism for BCG immunotherapy in bladder cancer. Nat Rev Urol. 2020:17:513–525. 10.1038/s41585-020-0346-4 32678343

[22] Atallah A et al Systemic versus localized Bacillus Calmette Guérin immunotherapy of bladder cancer promotes an anti-tumoral microenvironment: novel role of trained immunity. Int J Cancer. 2024:155:352–364. 10.1002/ijc.34897 38483404

[23] Wang Y, Wang GZ, Rabinovitch PS, Tabas I. Macrophage mitochondrial oxidative stress promotes atherosclerosis and nuclear factor-κB–mediated inflammation in macrophages. Circ Res. 2014:114:421–433. 10.1161/CIRCRESAHA.114.302153 24297735 PMC3946745

[24] Arts RJW et al Glutaminolysis and fumarate accumulation integrate immunometabolic and epigenetic programs in trained immunity. Cell Metab. 2016:24:807–819. 10.1016/j.cmet.2016.10.008 27866838 PMC5742541

[25] Netea MG et al Defining trained immunity and its role in health and disease. Nat Rev Immunol. 2020:20:375–388. 10.1038/s41577-020-0285-6 32132681 PMC7186935

[26] Keating ST et al The Set7 lysine methyltransferase regulates plasticity in oxidative phosphorylation necessary for trained immunity induced by β-glucan. Cell Rep. 2020:31:107548. 10.1016/j.celrep.2020.107548 32320649 PMC7184679

[27] Wang X et al MLL1, a histone H3K4 methyltransferase, regulates the expression of TNFα-mediated NF-κB downstream genes. J Cell Sci. 2012:125:4058–4066. 10.1242/jcs.103531 22623725

[28] Cai H et al Lactate activates trained immunity by fueling the tricarboxylic acid cycle and regulating histone lactylation. Nat Commun. 2025:16:3230. 10.1038/s41467-025-58563-2 40185732 PMC11971257

[29] Ziogas A et al Long-term histone lactylation connects metabolic and epigenetic rewiring in innate immune memory. Cell. 2025:188:2992–3012.e16. 10.1016/j.cell.2025.03.048 40318634

[30] Bannister S et al Neonatal BCG vaccination is associated with a long-term DNA methylation signature in circulating monocytes. Sci Adv. 2022:8:eabn4002. 10.1126/sciadv.abn4002 35930640 PMC9355358

[31] Qi C et al Long-term DNA methylation changes mediate heterologous cytokine responses after BCG vaccination. Genome Biol. 2025:26:180. 10.1186/s13059-025-03611-9 40629459 PMC12239379

[32] Fanucchi S et al Immune genes are primed for robust transcription by proximal long noncoding RNAs located in nuclear compartments. Nat Genet. 2019:51:138–150. 10.1038/s41588-018-0298-2 30531872

[33] Cheng S-C et al mTOR- and HIF-1α-mediated aerobic glycolysis as metabolic basis for trained immunity. Science (New York, N.Y.). 2014:345:1250684. 10.1126/science.1250684 25258083 PMC4226238

[34] Hao D et al Metabolic adaptations driving innate immune memory: mechanisms and therapeutic implications. J Leukoc Biol. 2025:117:qiaf037. 10.1093/jleuko/qiaf037 40138361 PMC12094284

[35] Bekkering S et al Oxidized low-density lipoprotein induces long-term proinflammatory cytokine production and foam cell formation via epigenetic reprogramming of monocytes. Arterioscler Thromb Vasc Biol. 2014:34:1731–1738. 10.1161/ATVBAHA.114.303887 24903093

[36] Mills TS et al A distinct metabolic and epigenetic state drives trained immunity in HSC-derived macrophages from autoimmune mice. Cell Stem Cell. 2024:31:1630–1649.e8. 10.1016/j.stem.2024.09.010 39413777 PMC11560650

[37] Tran BT, Jeyanathan V, Cao R, Kaufmann E, King KY. Hematopoietic stem and progenitor cells as a reservoir for trained immunity. eLife. 2025:14:e106610. 10.7554/eLife.106610 40905954 PMC12410968

[38] Baldridge MT, King KY, Boles NC, Weksberg DC, Goodell MA. Quiescent haematopoietic stem cells are activated by IFN-γ in response to chronic infection. Nature. 2010:465:793–797. 10.1038/nature09135 20535209 PMC2935898

[39] Nagai Y et al Toll-like receptors on hematopoietic progenitor cells stimulate innate immune system replenishment. Immunity. 2006:24:801–812. 10.1016/j.immuni.2006.04.008 16782035 PMC1626529

[40] Essers MAG et al IFNα activates dormant haematopoietic stem cells in vivo. Nature. 2009:458:904–908. 10.1038/nature07815 19212321

[41] Kain BN et al Hematopoietic stem and progenitor cells confer cross-protective trained immunity in mouse models. iScience. 2023:26:107596. 10.1016/j.isci.2023.107596 37664586 PMC10470378

[42] Damani-Yokota P, Khanna KM. Innate immune memory: the evolving role of macrophages in therapy. eLife. 2025:14:e108276. 10.7554/eLife.108276 41236793 PMC12618008

[43] Pietras EM et al Functionally distinct subsets of lineage-biased multipotent progenitors control blood production in normal and regenerative conditions. Cell Stem Cell. 2015:17:35–46. 10.1016/j.stem.2015.05.003 26095048 PMC4542150

[44] Ochando J, Mulder WJM, Madsen JC, Netea MG, Duivenvoorden R. Trained immunity—basic concepts and contributions to immunopathology. Nat Rev Nephrol. 2023:19:23–37. 10.1038/s41581-022-00633-5 36253509 PMC9575643

[45] Moorlag SJCFM et al β-Glucan induces protective trained immunity against Mycobacterium tuberculosis infection: a key role for IL-1. Cell Rep. 2020:31:107634. 10.1016/j.celrep.2020.107634 32433977 PMC7242907

[46] Lee A et al BCG vaccination stimulates integrated organ immunity by feedback of the adaptive immune response to imprint prolonged innate antiviral resistance. Nat Immunol. 2024:25:41–53. 10.1038/s41590-023-01700-0 38036767 PMC10932731

[47] Trzebanski S et al Classical monocyte ontogeny dictates their functions and fates as tissue macrophages. Immunity. 2024:57:1225–1242.e6. 10.1016/j.immuni.2024.04.019 38749446

[48] Guilliams M, Mildner A, Yona S. Developmental and functional heterogeneity of monocytes. Immunity. 2018:49:595–613. 10.1016/j.immuni.2018.10.005 30332628

[49] Hettinger J et al Origin of monocytes and macrophages in a committed progenitor. Nat Immunol. 2013:14:821–830. 10.1038/ni.2638 23812096

[50] Li S, Yao J-C, Li JT, Schmidt AP, Link DC. TLR7/8 agonist treatment induces an increase in bone marrow resident dendritic cells and hematopoietic progenitor expansion and mobilization. Exp Hematol. 2021:96:35–43.e7. 10.1016/j.exphem.2021.02.001 33556431 PMC9900459

[51] Teh YC, Ding JL, Ng LG, Chong SZ. Capturing the fantastic voyage of monocytes through time and space. Front Immunol. 2019:10:834. 10.3389/fimmu.2019.00834 31040854 PMC6476989

[52] Moorlag SJCFM et al Multi-omics analysis of innate and adaptive responses to BCG vaccination reveals epigenetic cell states that predict trained immunity. Immunity. 2024:57:171–187.e14. 10.1016/j.immuni.2023.12.005 38198850

[53] Chen J et al BCG-induced trained immunity: history, mechanisms and potential applications. J Transl Med. 2023:21:106. 10.1186/s12967-023-03944-8 36765373 PMC9913021

[54] de Araujo ACVSC, de Queiroz NMGP, Marinho FV, Oliveira SC. Bacillus Calmette-Guérin-trained macrophages elicit a protective inflammatory response against the pathogenic Bacteria *Brucella abortus*. J Immunol (Baltimore, Md.: 1950). 2023:211:791–803. 10.4049/jimmunol.2200642 PMC10530434 37477668

[55] Naqvi N et al BCG's role in strengthening immune responses: implications for tuberculosis and comorbid diseases. Infect Genet Evol. 2025:127:105703. 10.1016/j.meegid.2024.105703 39667418

[56] Foster M et al BCG-induced protection against *Mycobacterium tuberculosis* infection: evidence, mechanisms, and implications for next-generation vaccines. Immunol Rev. 2021:301:122–144. 10.1111/imr.12965 33709421 PMC8252066

[57] Zhang Y, Zhang Z, Tu C, Chen X, He R. Advanced glycation end products in disease development and potential interventions. Antioxidants. 2025:14:492. 10.3390/antiox14040492 40298887 PMC12024296

[58] Wculek SK, Dunphy G, Heras-Murillo I, Mastrangelo A, Sancho D. Metabolism of tissue macrophages in homeostasis and pathology. Cell Mol Immunol. 2022:19:384–408. 10.1038/s41423-021-00791-9 34876704 PMC8891297

[59] Hunter M et al Survival of monocytes and macrophages and their role in health and disease. Front Biosci (Landmark Edition). 2009:14:4079–4102. 10.2741/3514 PMC3708298 19273336

[60] Italiani P, Boraschi D. From monocytes to M1/M2 macrophages: phenotypical vs. functional differentiation. Front Immunol. 2014:5:514. 10.3389/fimmu.2014.00514 25368618 PMC4201108

[61] Cormican S, Griffin MD. Human monocyte subset distinctions and function: insights from gene expression analysis. Front Immunol. 2020:11:1070. 10.3389/fimmu.2020.01070 32582174 PMC7287163

[62] Chavakis T, Wielockx B, Hajishengallis G. Inflammatory modulation of hematopoiesis: linking trained immunity and clonal hematopoiesis with chronic disorders. Annu Rev Physiol. 2022:84:183–207. 10.1146/annurev-physiol-052521-013627 34614373

[63] Pradhan K, Yi Z, Geng S, Li L. Development of exhausted memory monocytes and underlying mechanisms. Front Immunol. 2021:12:778830. 10.3389/fimmu.2021.778830 34777396 PMC8583871

[64] Schrijver DP et al Resolving sepsis-induced immunoparalysis via trained immunity by targeting interleukin-4 to myeloid cells. Nat Biomed Eng. 2023:7:1097–1112. 10.1038/s41551-023-01050-0 37291433 PMC10504080

[65] Jeljeli MM, Adamopoulos IE. Innate immune memory in inflammatory arthritis. Nat Rev Rheumatol. 2023:19:627–639. 10.1038/s41584-023-01009-0 37674048 PMC10721491

[66] Robinson KA, Akbar N, Baidžajevas K, Choudhury RP. Trained immunity in diabetes and hyperlipidemia: emerging opportunities to target cardiovascular complications and design new therapies. FASEB J. 2023:37:e23231. 10.1096/fj.202301078R 37779347 PMC10947360

[67] Kim HY, Lee W-W. Trained immunity induced by DAMPs and LAMPs in chronic inflammatory diseases. Exp Mol Med. 2025:57:2137–2147. 10.1038/s12276-025-01542-w 41028520 PMC12586657

[68] Ginhoux F, Guilliams M. Tissue-resident macrophage ontogeny and homeostasis. Immunity. 2016:44:439–449. 10.1016/j.immuni.2016.02.024 26982352

[69] Levy M, Kolodziejczyk AA, Thaiss CA, Elinav E. Dysbiosis and the immune system. Nat Rev Immunol. 2017:17:219–232. 10.1038/nri.2017.7 28260787

[70] Stothers CL, Luan L, Fensterheim BA, Bohannon JK. Hypoxia-inducible factor-1α regulation of myeloid cells. J Mol Med (Berlin, Germany). 2018:96:1293–1306. 10.1007/s00109-018-1710-1 PMC6292431 30386909

[71] Aegerter H, Lambrecht BN, Jakubzick CV. Biology of lung macrophages in health and disease. Immunity. 2022:55:1564–1580. 10.1016/j.immuni.2022.08.010 36103853 PMC9533769

[72] Wang T et al Influenza-trained mucosal-resident alveolar macrophages confer long-term antitumor immunity in the lungs. Nat Immunol. 2023:24:423–438. 10.1038/s41590-023-01428-x 36807642

[73] Yao Y et al Induction of autonomous memory alveolar macrophages requires T cell help and is critical to trained immunity. Cell. 2018:175:1634–1650.e17. 10.1016/j.cell.2018.09.042 30433869

[74] Zazara DE, Belios I, Lücke J, Zhang T, Giannou AD. Tissue-resident immunity in the lung: a first-line defense at the environmental interface. Semin Immunopathol. 2022:44:827–854. 10.1007/s00281-022-00964-2 36305904 PMC9614767

[75] Zahalka S et al Trained immunity of alveolar macrophages requires metabolic rewiring and type 1 interferon signaling. Mucosal Immunol. 2022:15:896–907. 10.1038/s41385-022-00528-5 35856089 PMC9385480

[76] Idiiatullina E, Parker D. Trained immunity in the lung. eLife. 2025:14:e104918. 10.7554/eLife.104918 40748050 PMC12316460

[77] Hernandez A et al INTRAPULMONARY TREATMENT WITH A NOVEL TLR4 AGONIST CONFERS PROTECTION AGAINST KLEBSIELLA PNEUMONIA. Shock (Augusta, Ga.). 2022:58:295–303. 10.1097/SHK.0000000000001977 36018281 PMC9647733

[78] Chakraborty S et al Trained immunity of alveolar macrophages enhances injury resolution via KLF4-MERTK-mediated efferocytosis. J Exp Med. 2023:220:e20221388. 10.1084/jem.20221388 37615937 PMC10450795

[79] Prevel R et al β-Glucan reprograms alveolar macrophages via neutrophil/IFNγ axis in a murine model of lung injury. eLife. 2025:13:RP102068. 10.7554/eLife.102068 40624927 PMC12237401

[80] Jiao Y, Wu L, Huntington ND, Zhang X. Crosstalk between gut microbiota and innate immunity and its implication in autoimmune diseases. Front Immunol. 2020:11:282. 10.3389/fimmu.2020.00282 32153586 PMC7047319

[81] Velikova T et al Mucosal immunity and trained innate immunity of the gut. Gastroenterol Insights. 2024:15:661–675. 10.3390/gastroent15030048

[82] Pellon A, Palacios A, Abecia L, Rodríguez H, Anguita J. Friends to remember: innate immune memory regulation by the microbiota. Trends Microbiol. 2025:33:510–520. 10.1016/j.tim.2024.12.002 39794207

[83] Schulthess J et al The short chain fatty acid butyrate imprints an antimicrobial program in macrophages. Immunity. 2019:50:432–445.e7. 10.1016/j.immuni.2018.12.018 30683619 PMC6382411

[84] Chang PV, Hao L, Offermanns S, Medzhitov R. The microbial metabolite butyrate regulates intestinal macrophage function via histone deacetylase inhibition. Proc Natl Acad Sci U S A. 2014:111:2247–2252. 10.1073/pnas.1322269111 24390544 PMC3926023

[85] Arpaia N, Rudensky AY. Microbial metabolites control gut inflammatory responses. Proc Natl Acad Sci U S A. 2014:111:2058–2059. 10.1073/pnas.1323183111 24434557 PMC3926042

[86] Ji J et al Microbial metabolite butyrate facilitates M2 macrophage polarization and function. Sci Rep. 2016:6:24838. 10.1038/srep24838 27094081 PMC4837405

[87] Martin-Gallausiaux C, Marinelli L, Blottière HM, Larraufie P, Lapaque N. SCFA: mechanisms and functional importance in the gut. Proc Nutr Soc. 2021:80:37–49. 10.1017/S0029665120006916 32238208

[88] Chapin N, Fernandez J, Poole J, Delatte B. Anchor-based bisulfite sequencing determines genome-wide DNA methylation. Commun Biol. 2022:5:596. 10.1038/s42003-022-03543-1 35710818 PMC9203462

[89] Walachowski S et al Oral supplementation with yeast β-glucans improves the resolution of Escherichia coli-associated inflammatory responses independently of monocyte/macrophage immune training. Front Immunol. 2022:13:1086413. 10.3389/fimmu.2022.1086413 36605196 PMC9809295

[90] Hayakawa K, Nishitani K, Tanaka S. Kynurenine, 3-OH-kynurenine, and anthranilate are nutrient metabolites that alter H3K4 trimethylation and H2AS40 O-GlcNAcylation at hypothalamus-related loci. Sci Rep. 2019:9:19768. 10.1038/s41598-019-56341-x 31875008 PMC6930210

[91] Tiszlavicz Z et al Different inhibitory effects of kynurenic acid and a novel kynurenic acid analogue on tumour necrosis factor-α (TNF-α) production by mononuclear cells, HMGB1 production by monocytes and HNP1-3 secretion by neutrophils. Naunyn Schmiedebergs Arch Pharmacol. 2011:383:447–455. 10.1007/s00210-011-0605-2 21336543

[92] Salimi Elizei S, Poormasjedi-Meibod M-S, Wang X, Kheirandish M, Ghahary A. Kynurenic acid downregulates IL-17/1L-23 axis in vitro. Mol Cell Biochem. 2017:431:55–65. 10.1007/s11010-017-2975-3 28285360

[93] Sadok I, Jędruchniewicz K. Dietary kynurenine pathway metabolites-source, fate, and chromatographic determinations. Int J Mol Sci. 2023:24:16304. 10.3390/ijms242216304 38003492 PMC10671297

[94] Taylor CT, Scholz CC. The effect of HIF on metabolism and immunity. Nat Rev Nephrol. 2022:18:573–587. 10.1038/s41581-022-00587-8 35726016 PMC9208707

[95] Sato T, Takeda N. The roles of HIF-1α signaling in cardiovascular diseases. J Cardiol. 2023:81:202–208. 10.1016/j.jjcc.2022.09.002 36127212

[96] Tannahill GM et al Succinate is an inflammatory signal that induces IL-1β through HIF-1α. Nature. 2013:496:238–242. 10.1038/nature11986 23535595 PMC4031686

[97] Zhang S, Zhang Y, Duan X, Wang B, Zhan Z. Targeting NPM1 epigenetically promotes postinfarction cardiac repair by reprogramming reparative macrophage metabolism. Circulation. 2024:149:1982–2001. 10.1161/CIRCULATIONAHA.123.065506 38390737 PMC11175795

[98] Lavine KJ et al The macrophage in cardiac homeostasis and disease. J Am Coll Cardiol. 2018:72:2213–2230. 10.1016/j.jacc.2018.08.2149 30360829 PMC6209119

[99] Seijkens T et al Hypercholesterolemia-induced priming of hematopoietic stem and progenitor cells aggravates atherosclerosis. FASEB J. 2014:28:2202–2213. 10.1096/fj.13-243105 24481967

[100] Murphy AJ et al Apoe regulates hematopoietic stem cell proliferation, monocytosis, and monocyte accumulation in atherosclerotic lesions in mice. J Clin Invest. 2011:121:4138–4149. 10.1172/JCI57559 21968112 PMC3195472

[101] Van Kampen E, Jaminon A, Van Berkel TJC, Van Eck M. Diet-induced (epigenetic) changes in bone marrow augment atherosclerosis. J Leukoc Biol. 2014:96:833–841. 10.1189/jlb.1A0114-017R 25024399

[102] Lavillegrand J-R et al Alternating high-fat diet enhances atherosclerosis by neutrophil reprogramming. Nature. 2024:634:447–456. 10.1038/s41586-024-07693-6 39232165 PMC12019644

[103] Christ A et al Western diet triggers NLRP3-dependent innate immune reprogramming. Cell. 2018:172:162–175.e14. 10.1016/j.cell.2017.12.013 29328911 PMC6324559

[104] Pirillo A, Norata GD, Catapano AL. LOX-1, OxLDL, and atherosclerosis. Mediators Inflamm. 2013:2013:152786. 10.1155/2013/152786 23935243 PMC3723318

[105] Lim KRQ et al Toll-like receptor 4 induces trained innate immune tolerance in the heart in a model of stress-induced cardiomyopathy. Cardiovasc Res. 2025:121:2055–2069. 10.1093/cvr/cvaf158 40924855 PMC12560765

[106] Yamamoto T et al Cardiac Sirt1 mediates the cardioprotective effect of caloric restriction by suppressing local complement system activation after ischemia-reperfusion. Am J Physiol Heart Circ Physiol. 2016:310:H1003–H1014. 10.1152/ajpheart.00676.2015 26873964

[107] Li X et al Inhibition of fatty acid oxidation enables heart regeneration in adult mice. Nature. 2023:622:619–626. 10.1038/s41586-023-06585-5 37758950 PMC10584682

[108] Schüttler D, Clauss S, Weckbach LT, Brunner S. Molecular mechanisms of cardiac remodeling and regeneration in physical exercise. Cells. 2019:8:1128. 10.3390/cells8101128 31547508 PMC6829258

[109] Byrd AL, Belkaid Y, Segre JA. The human skin microbiome. Nat Rev Microbiol. 2018:16:143–155. 10.1038/nrmicro.2017.157 29332945

[110] Linehan JL et al Non-classical immunity controls microbiota impact on skin immunity and tissue repair. Cell. 2018:172:784–796.e18. 10.1016/j.cell.2017.12.033 29358051 PMC6034182

[111] Carlile SR et al Staphylococcus aureus induced trained immunity in macrophages confers heterologous protection against gram-negative bacterial infection. iScience. 2024:27:111284. 10.1016/j.isci.2024.111284 39618498 PMC11607596

[112] Severn MM, Horswill AR. Staphylococcus epidermidis and its dual lifestyle in skin health and infection. Nat Rev Microbiol. 2023:21:97–111. 10.1038/s41579-022-00780-3 36042296 PMC9903335

[113] Landemaine L et al Staphylococcus epidermidis isolates from atopic or healthy skin have opposite effect on skin cells: potential implication of the AHR pathway modulation. Front Immunol. 2023:14:1098160. 10.3389/fimmu.2023.1098160 37304256 PMC10250813

[114] Belkaid Y, Tamoutounour S. The influence of skin microorganisms on cutaneous immunity. Nat Rev Immunol. 2016:16:353–366. 10.1038/nri.2016.48 27231051

[115] Gallo RL . S. epidermidis influence on host immunity: more than skin deep. Cell Host Microbe. 2015:17:143–144. 10.1016/j.chom.2015.01.012 25674978 PMC5103695

[116] Nakayama Y, Fujiu K. Innate immune memory in macrophage differentiation and cardiovascular diseases. Inflamm Regen. 2025:45:17. 10.1186/s41232-025-00382-5 40462201 PMC12131520

[117] Lin X-Q et al Trained immunity in recurrent Staphylococcus aureus infection promotes bacterial persistence. PLoS Pathog. 2024:20:e1011918. 10.1371/journal.ppat.1011918 38241414 PMC10798626

[118] Williams MR et al Interplay of Staphylococcal and host proteases promotes skin barrier disruption in Netherton syndrome. Cell Rep. 2020:30:2923–2933.e7. 10.1016/j.celrep.2020.02.021 32130897 PMC7183042

[119] Yoshida K et al High-dose radiation preferentially induces the clonal expansion of hematopoietic progenitor cells over mature T and B cells in mouse bone marrow. Stem Cell Reports. 2025:20:102423. 10.1016/j.stemcr.2025.102423 40020684 PMC11960520

[120] Kim H-Y et al UV-induced DNA methyltransferase 1 promotes hypermethylation of tissue inhibitor of metalloproteinase 2 in the human skin. J Dermatol Sci. 2018:91:19–27. 10.1016/j.jdermsci.2018.03.009 29685765

[121] Becklund BR, Severson KS, Vang SV, DeLuca HF. UV radiation suppresses experimental autoimmune encephalomyelitis independent of vitamin D production. Proc Natl Acad Sci U S A. 2010:107:6418–6423. 10.1073/pnas.1001119107 20308557 PMC2851981

[122] Guan Y, Yang YJ, Nagarajan P, Ge Y. Transcriptional and signalling regulation of skin epithelial stem cells in homeostasis, wounds and cancer. Exp Dermatol. 2021:30:529–545. 10.1111/exd.14247 33249665 PMC8016706

[123] Tang X, Yang T, Yu D, Xiong H, Zhang S. Current insights and future perspectives of ultraviolet radiation (UV) exposure: friends and foes to the skin and beyond the skin. Environ Int. 2024:185:108535. 10.1016/j.envint.2024.108535 38428192

[124] Naik S et al Inflammatory memory sensitizes skin epithelial stem cells to tissue damage. Nature. 2017:550:475–480. 10.1038/nature24271 29045388 PMC5808576

[125] Guerrero P et al GM-CSF receptor expression determines opposing innate memory phenotypes at different stages of myelopoiesis. Blood. 2024:143:2763–2777. 10.1182/blood.2024024330 38603633 PMC11251219

[126] Van Puffelen JH et al Intravesical BCG in patients with non-muscle invasive bladder cancer induces trained immunity and decreases respiratory infections. J Immunother Cancer. 2023:11:e005518. 10.1136/jitc-2022-005518 36693678 PMC9884868

[127] Koushki K et al Anti-inflammatory action of statins in cardiovascular disease: the role of inflammasome and toll-like receptor pathways. Clin Rev Allergy Immunol. 2021:60:175–199. 10.1007/s12016-020-08791-9 32378144 PMC7985098

[128] Wang S et al Statins attenuate activation of the NLRP3 inflammasome by oxidized LDL or TNFα in vascular endothelial cells through a PXR-dependent mechanism. Mol Pharmacol. 2017:92:256–264. 10.1124/mol.116.108100 28546421

[129] Leung YY, Yao Hui LL, Kraus VB. Colchicine—update on mechanisms of action and therapeutic uses. Semin Arthritis Rheum. 2015:45:341–350. 10.1016/j.semarthrit.2015.06.013 26228647 PMC4656054

[130] Pinkosky SL et al Liver-specific ATP-citrate lyase inhibition by bempedoic acid decreases LDL-C and attenuates atherosclerosis. Nat Commun. 2016:7:13457. 10.1038/ncomms13457 27892461 PMC5133702

[131] Hernandez A et al Pretreatment with a novel toll-like receptor 4 agonist attenuates renal ischemia-reperfusion injury. Am J Physiol Renal Physiol. 2023:324:F472–F482. 10.1152/ajprenal.00248.2022 36995924 PMC10151043

[132] Sudduth ER, Trautmann-Rodriguez M, Gill N, Bomb K, Fromen CA. Aerosol pulmonary immune engineering. Adv Drug Deliv Rev. 2023:199:114831. 10.1016/j.addr.2023.114831 37100206 PMC10527166

[133] Gallotta M et al Inhaled TLR9 agonist renders lung tumors permissive to PD-1 blockade by promoting optimal CD4+ and CD8+ T-cell interplay. Cancer Res. 2018:78:4943–4956. 10.1158/0008-5472.CAN-18-0729 accessed 2025 Dec 11. 29945961

[134] Kell SA et al Preclinical development of the TLR9 agonist DV281 as an inhaled aerosolized immunotherapeutic for lung cancer: pharmacological profile in mice, non-human primates, and human primary cells. Int Immunopharmacol. 2019:66:296–308. 10.1016/j.intimp.2018.11.019 30502651

[135] Kan S et al TLR7 agonist loaded airway epithelial targeting nanoparticles stimulate innate immunity and suppress viral replication in human bronchial epithelial cells. Int J Pharm. 2022:617:121586. 10.1016/j.ijpharm.2022.121586 35181464

[136] Wang RX, Lee JS, Campbell EL, Colgan SP. Microbiota-derived butyrate dynamically regulates intestinal homeostasis through regulation of actin-associated protein synaptopodin. Proc Nat Acad Sci U S A. 2020:117:11648–11657. 10.1073/pnas.1917597117 PMC7260972 32398370

[137] Heilborn JD, Weber G, Grönberg A, Dieterich C, Ståhle M. Topical treatment with the vitamin D analogue calcipotriol enhances the upregulation of the antimicrobial protein hCAP18/LL-37 during wounding in human skin *in vivo*. Exp Dermatol. 2010:19:332–338. 10.1111/j.1600-0625.2009.00997.x 19878298

[138] Azin M, Ngo KH, Hojanazarova J, Demehri S. Topical calcipotriol plus imiquimod immunotherapy for nonkeratinocyte skin cancers. JID Innov. 2023:3:100221. 10.1016/j.xjidi.2023.100221 37731472 PMC10507651

[139] Kartha VK et al Functional inference of gene regulation using single-cell multi-omics. Cell Genom. 2022:2:100166. 10.1016/j.xgen.2022.100166 36204155 PMC9534481

[140] Troutman TD, Kofman E, Glass CK. Exploiting dynamic enhancer landscapes to decode macrophage and microglia phenotypes in health and disease. Mol Cell. 2021:81:3888–3903. 10.1016/j.molcel.2021.08.004 34464593 PMC8500948

[141] Liu W et al Enhanced cardiomyocyte NLRP3 inflammasome-mediated pyroptosis promotes d-galactose–induced cardiac aging. J Am Heart Assoc. 2024:13:e032904. 10.1161/JAHA.123.032904 38979831 PMC11292767

[142] Choi S et al Organoid modeling of lung-resident immune responses to SARS-CoV-2 infection. *2023*. 10.21203/rs.3.rs-2870695/v1

[143] Li T et al Immune organoids: emerging platforms for modeling and analyzing human adaptive immunity. Front Immunol. 2025:16:1632117. 10.3389/fimmu.2025.1632117 40843000 PMC12364649

[144] Papp D, Korcsmaros T, Hautefort I. Revolutionizing immune research with organoid-based co-culture and chip systems. Clin Exp Immunol. 2024:218:40–54. 10.1093/cei/uxae004 38280212 PMC11404127

[145] van Puffelen JH, Campbell C, Gander-Meisterernst I, Holldack J, Lukey PT. Trained immunity: RoadMap for drug discovery and development. eLife. 2025:14:e108465. 10.7554/eLife.108465 41432375 PMC12726827

[146] Xu W et al Reversing inflammatory diseases via trained immunity: mechanisms, challenges, and prospects. Front Immunol. 2025:16:1666233. 10.3389/fimmu.2025.1666233 41169375 PMC12568716

